# transXpress: a Snakemake pipeline for streamlined de novo transcriptome assembly and annotation

**DOI:** 10.1186/s12859-023-05254-8

**Published:** 2023-04-04

**Authors:** Timothy R. Fallon, Tereza Čalounová, Martin Mokrejš, Jing-Ke Weng, Tomáš Pluskal

**Affiliations:** 1 Scripps Institution of Oceanography, UC San Diego, 9500 Gilman Dr, La Jolla, CA 92093 USA; 2 Institute of Organic Chemistry and Biochemistry of the Czech Academy of Sciences, Flemingovo náměstí 2, 16000 Prague 6, Czech Republic; 3 Whitehead Institute for Biomedical Research, 455 Main Street, Cambridge, MA 02142 USA; 4 Department of Biology, Massachusetts Institute of Technology, Cambridge, MA 02139 USA

**Keywords:** De novo transcriptome assembly, RNA-seq, Non-model organisms, Transcriptome annotation, Differential expression analysis, Reproducible software, High-performance computing

## Abstract

**Background:**

RNA-seq followed by de novo transcriptome assembly has been a transformative technique in biological research of non-model organisms, but the computational processing of RNA-seq data entails many different software tools. The complexity of these de novo transcriptomics workflows therefore presents a major barrier for researchers to adopt best-practice methods and up-to-date versions of software.

**Results:**

Here we present a streamlined and universal de novo transcriptome assembly and annotation pipeline, transXpress, implemented in Snakemake. transXpress supports two popular assembly programs, Trinity and rnaSPAdes, and allows parallel execution on heterogeneous cluster computing hardware.

**Conclusions:**

transXpress simplifies the use of best-practice methods and up-to-date software for de novo transcriptome assembly, and produces standardized output files that can be mined using SequenceServer to facilitate rapid discovery of new genes and proteins in non-model organisms.

## Background

De novo transcriptome assembly of short-read RNA-seq data followed by prediction of open reading frames (ORFs) and automated annotation of predicted proteins is widely used for studying non-model eukaryotic organisms without a reference genome [1, 2]. The NCBI Sequence Read Archive (SRA) database currently contains over 3 million RNA-seq datasets, including hundreds of thousands from non-model eukaryotes [3]. These datasets represent a rich and continuously growing resource for diverse biological research across the tree of life. In contrast, only ~ 6900 eukaryotic transcriptome assemblies have been uploaded to the NCBI Transcriptome Shotgun Assembly (TSA) database to date, reflecting the difficulties in producing and uploading high-quality assemblies [4]. Generating and annotating a de novo transcriptome assembly requires numerous bioinformatic tools that can be difficult to install, and best practices are not always followed [5].

We surveyed existing pipelines for RNA-seq data analysis, including de novo transcriptome assembly and gene annotation tasks (Table 1). To date, four pipelines have been published for de novo transcriptome assembly, two of which (Rnnotator [6] and themira [7]) have been discontinued since their publication. Several other pipelines are available for aligning RNA-seq reads to a reference genome. Only a few of them support alignment of raw reads to a de novo assembled or reference transcriptome, depending mostly on the read aligner used. However, such pipelines generally were not designed to assist with gene discovery in non-model organisms. Presently, Pincho [8] is the only maintained pipeline that supports both de novo transcriptome assembly and transcript annotation using a variety of tools. However, Pincho does not support distributed computing on high-performance computational clusters (HPCs), and therefore has limited utility for processing large sequencing datasets.

**Table 1:** Overview of existing pipelines for RNA-seq data analysis

Pipeline	Platform	Preprocessing	Assembly	Read mapping	Expression analysis	Functional annotation
transXpress	Snakemake	trimmomatic, FastQC, MultiQC	Trinity, rnaSPAdes	bowtie2 (optional)	kallisto, edgeR	BLAST, TargetP, SignalP, TMHMM, BUSCO
Pincho [8]	Bash, python3	trimmomatic, Rcorrector, TransRate, CD-HIT	Trinity, rnaSPAdes, BinPacker, IDBA-tran, Velvet-Oases, Shannon, Trans-AbySS, TransLig	HISAT2	kallisto, RSEM	BLAST, BUSCO, TransRate
RNAflow [9]	Nextflow	FastQC, MultiQC, fastp, SortMeRNA	Trinity	HISAT2	DESeq2	BUSCO, dammit
Rnnotator (unavailable) [6]	Unknown		Velvet, AMOS			
themira (unavailable) [7]	Unknown	FastXtoolkit, FastX, CAP3	Velvet-Oases			Blast2GO
nf-core/rnaseq [10]	Nextflow	FastQC, TrimGalore	(None)	STAR, HISAT2	RSEM, Salmon, DESeq2	
Pipeliner [11]	Nextflow	FastQC, MultiQC, TrimGalore	(None)	STAR, HISAT2	StringTie, HTSeq, featureCounts	
VIPER [12]	Snakemake	RSeQC	(None)	STAR	Picard, Cufflinks, RSeQC, ComBat, DESeq2, PCA	VarScan, Gostats, GAGE, Pathview, ClusterProfiler, STAR-fusion, TRUST, TIMER, virus contamination detection
RASflow [13]	Snakemake	TrimGalore, FastQC, MultiQC	(None)	Salmon, HiSAT2	featureCounts or htseq-count, Qualimap, edgeR, DESeq2	
hppRNA [14]	Snakemake	cutadapt, FastQC, PRINSEQ, FASTX-toolkit	(None)	Tophat, bowtie, subread, STAR, HiSAT	Cufflinks, featureCounts, RSEM, eXpress, kallisto, StringTie, ngs.plot, Cuffdiff, DESeq2, EBSeq, edgeR, sleuth, Ballgown	GATK, FusionCatcher
TRAPLINE [15]	Galaxy	FastxClipper, FastQC, FASTQ, FASTX-toolkit	(None)	Tophat, bowtie	Picard, Cufflinks, Cuffdiff	DAVID, miRanda, BioGRID
QuickRNAseq [16]	bash, Perl, R	RSeQC	(None)	STAR	featureCounts, RSeQC, edgeR	VarScan
ARMOR [17]	Snakemake	TrimGalore, FastQC + MultiQC	(None)	Salmon, STAR	edgeR, DRIMSeq	
BISR-RNAseq [18]	PBS, bash, shiny, R	FastQC + MultiQC	(None)	HiSAT2	Picard, featureCounts, RSeQC, limma, edgeR	
RNAseq123 [19]	Bioconductor		(None)		edgeR, limma, glimma	

The table summarizes the architecture and individual tools used in the pipelines for the main steps of data processing. Five of the pipelines (transXpress, Pincho, RNAflow, themira, Rnnotator) include a step of de novo transcriptome assembly, while the others require a reference genome or transcriptome

Here, we present a new de novo transcriptome assembly pipeline, transXpress, which streamlines reproducible assembly of transcripts, quantification of transcript expression levels, and gene and protein prediction and annotation. transXpress also supports parallel execution on heterogeneous cluster computing hardware.

## Implementation

### Workflow engine

Older RNA-seq pipelines were typically implemented as shell scripts with the use of Perl, Python or R to execute the relevant downstream analyses. Recently, there is a strong tendency to employ bioinformatic workflow engines such as Snakemake, Nextflow or Galaxy [20–22]. Owing to its general simplicity and ease of use, we selected Snakemake to handle the dependencies between the executed tasks, to avoid repeated computations upon pipeline re-execution, and to support cluster computing [20]. The users of transXpress are advised to install required dependencies using Conda [23] and Python’s PIP package management systems, as described on the transXpress GitHub page [24].

**Fig. 1:**
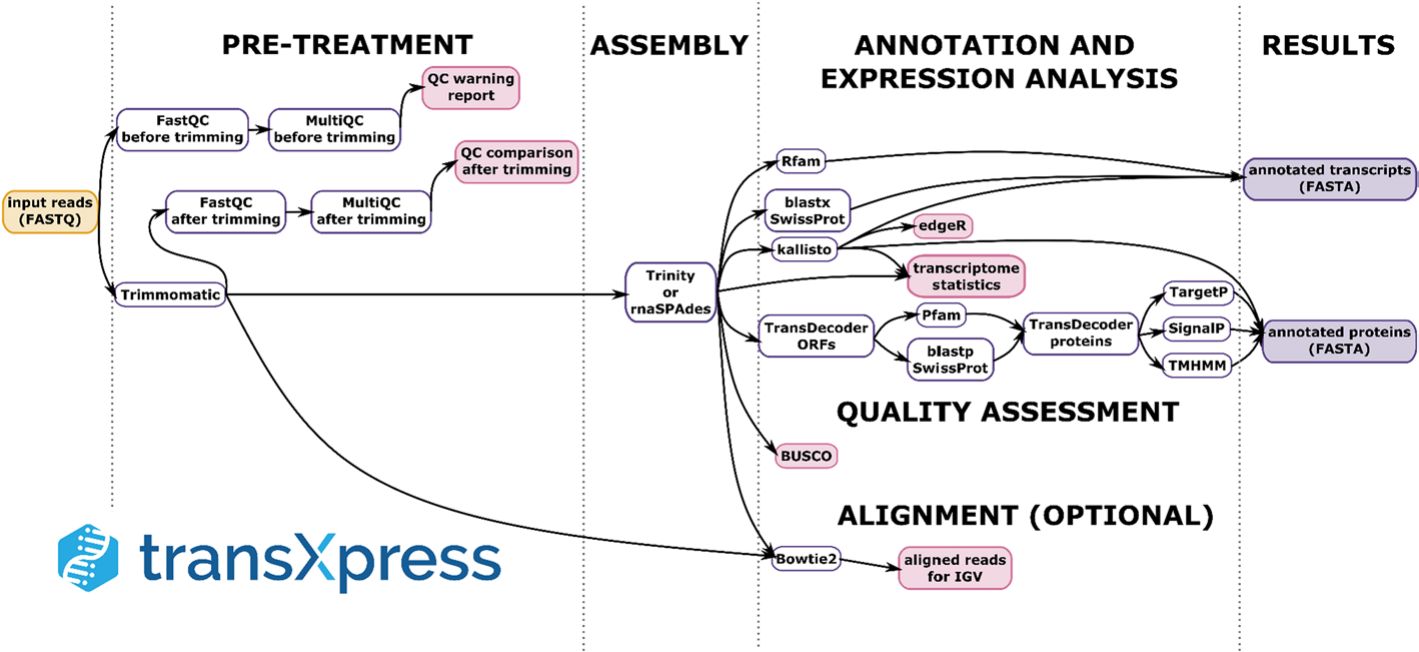
A schema of the data processing steps performed by the transXpress pipeline. The input data are on the very left in a yellow-colored frame. Initial data pre-treatment tasks are on the left, followed by assembly and tasks executed largely in parallel (annotation and expression analysis). Output data types are in a purple background on the very right. This is a manually simplified version of the directed acyclic graph (DAG) of Snakemake tasks. The DAG can be automatically generated by Snakemake for each transXpress run

The transXpress pipeline (Fig. 1) performs parallel execution of the underlying tools whenever possible. Furthermore, it splits the input datafiles (e.g., for the Trimmomatic and the FASTA annotation steps) into multiple partitions (batches) to speed up even single-threaded tasks by parallelization. The partial results files from such split tasks are then merged automatically back into a single output file. In the case of the Trinity assembler, the individual jobs generated within Trinity by the ‘Chrysalis’ phase as input for the ‘Butterfly’ phase, are automatically parallelized by transXpress [25, 26]. The output files from all the underlying tools, including their graphical results, are retained in the project folder.

### Data pre-treatment

The quality of the input sequencing reads has a major impact on the quality of the final transcriptome assembly [27]. To assess the quality of the provided reads, transXpress uses the FastQC tool [28]. Its wrapper add-on MultiQC [29] further aggregates and summarizes FastQC reports of all samples into a single report, providing an easy overview of the quality of sample preparation, library construction, and sequencing across all samples. Such a report is fundamental for the subsequent interpretation of the data.

Sequencing adapters and poor quality reads are removed using Trimmomatic [30]. Trimming the reads is very important for de novo assembly, since artificially introduced sequences (various types of adapters and their dimers, multimers, partial copies, or PCR-based artifacts) may interfere with the extension of contigs. After read trimming, transXpress performs another round of FastQC/MultiQC quality assessment and checks the generated report for potential warnings.

### de novo transcriptome assembly

Roughly ten de novo transcriptome assemblers for short RNA-seq reads have been developed and are in common use [31]. Among them, Trinity [25], rnaSPAdes [32] and TransAbyss [26], are the most widely used tools, and a recent evaluation indicated these three assemblers generally outperformed other tools [33]. All three utilize kmer-based De Bruijn graph assembly, which often requires a large amount of memory for the kmer frequency counting step. transXpress pools the sequencing reads for all provided samples and performs de novo assembly either using Trinity or rnaSPAdes, depending on the configuration settings provided by the user. Since these assemblers were primarily developed for high-quality short-read sequences, the range of supported sequencers includes Illumina, DNBSEQ, MGISEQ, or BGISEQ platforms, as well as older Roche/454 instruments [34]. transXpress does not support assembly from long-read sequencers such as PacBio or Nanopore. The assembled transcripts are further processed with TransDecoder [26] to identify likely protein-coding regions (ORFs). In case multiple potential ORFs are identified within a single transcript, TransDecoder reports all of them, leading to multiple protein sequences being subject to downstream annotation tasks in transXpress.

For each assembled transcriptome, transXpress reports simple statistics using scripts provided by the Trinity assembler (e.g., the number of assembled isoforms and genes, median contig length, contig Nx and ExN50 values) [35]. Further, transXpress runs the Benchmarking Universal Single-Copy Orthologs (BUSCO) tool to assess the completeness of the transcriptome by estimating completeness and redundancy in terms of expected gene content [36].

### Expression analysis and transcriptome annotation

The underlying RNA-seq reads used for the transcriptome assembly are also used to estimate transcript expression levels (transcript-per-million or TPM values) using kallisto, a fast alignment-free method for near-optimal expression quantification at the transcript isoform level [37]. As an optional step, full read-to-transcript local alignments can also be performed using Bowtie2 [38], to allow for troubleshooting and manual inspection of read coverage, for example in Integrated Genomics Viewer [39]. If multiple samples are included, transXpress performs differential expression analysis using edgeR [40]. This step also generates graphical output in the form of heat maps with hierarchical clustering analysis, using Perl and R scripts provided by the Trinity assembler [26]. The information about sample groups for differential expression analyses is obtained automatically from the transXpress main input file *samples.txt*, which defines the sample groups, replicates, and paths to raw sequencing reads (FASTQ files) for each sample.

The assembled transcriptome is further decorated with automated annotations. NCBI BLAST + [41] searches (blastx and blastp) are performed against the curated UniProtKB/Swiss-Prot database [42]; hmmer3 [43] is used to search through the Pfam-A database of protein domains [44]; and cmscan from the Infernal package [45] is used to search the Rfam database of non-coding RNA sequences [46]. Moreover, transXpress uses SignalP 6.0 and TargetP 2.0 to predict N-terminal signaling and targeting peptides [47, 48]. A Python re-implementation of the widely used TMHMM algorithm is employed for prediction of transmembrane helices [49].

The resulting flat files are parsed via custom Python scripts and the collected annotations are used to decorate the output FASTA files with transcripts and predicted protein coding sequences.

### Transcriptome mining

**Fig. 2:**
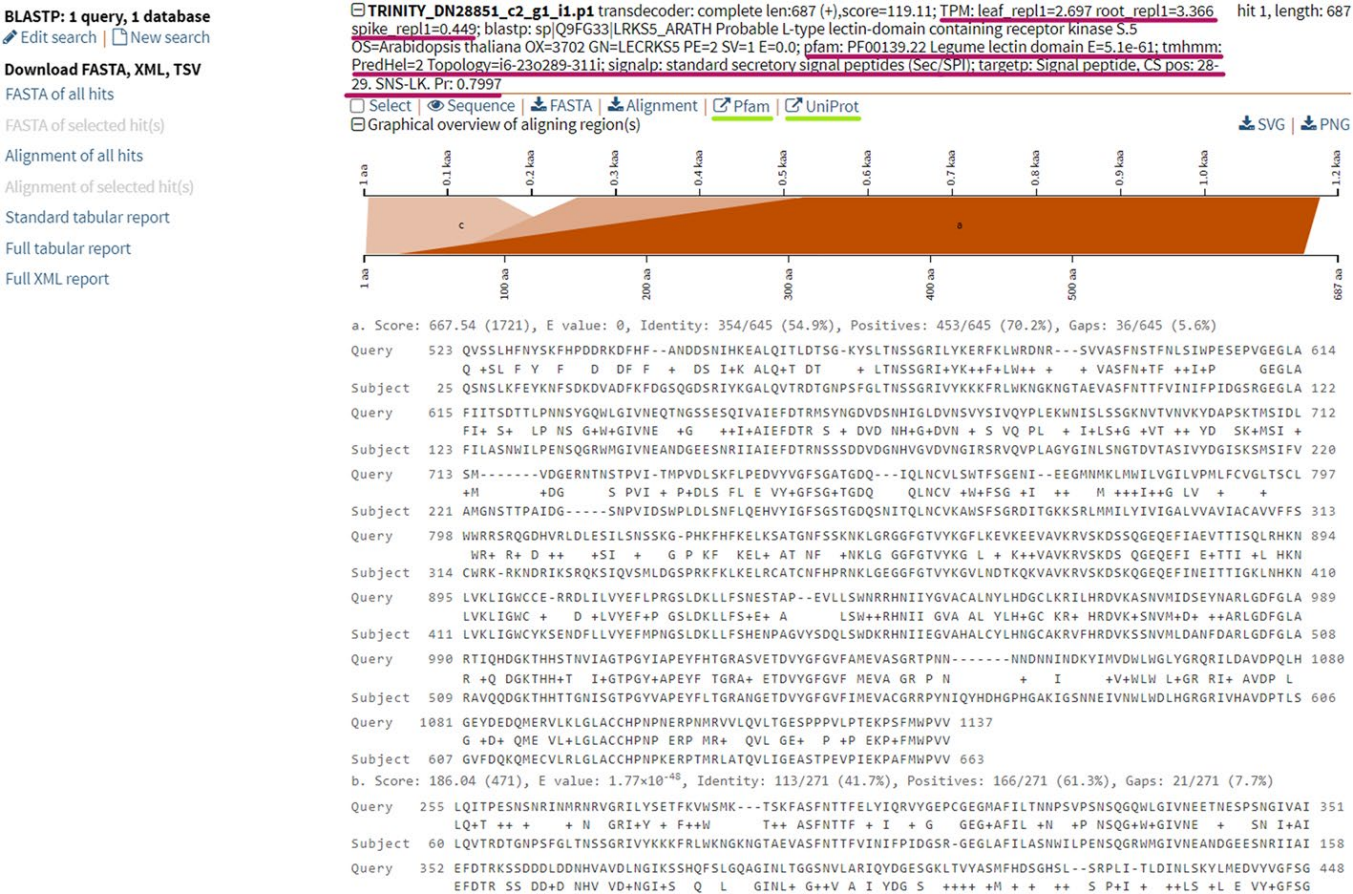
An example entry of a transcript annotated by the transXpress pipeline and rendered through SequenceServer 2.0.0 [50]. A number of annotations including TPM (expression quantification) values, protein domain and transmembrane domain predictions, subcellular localization, and signaling peptide predictions are annotated (underlined in purple). Auto-generated external hyperlinks are added as well (underlined in green). The example protein sequence was shortened for clarity

The most user-friendly way to mine the annotated FASTA files generated by transXpress is to use SequenceServer [50], which enables performing BLAST + [51] searches against custom FASTA sequence databases. For every hit, SequenceServer displays its alignment to the query and also the FASTA headers of each sequence, which include functional annotations created with transXpress—expression levels in different samples, the best BLAST hit in SwissProt, identified Pfam domains, topology prediction for transmembrane proteins, subcellular localization and prediction of targeting peptides, and auto-generated external hyperlinks to relevant Pfam and UniProt entries (Fig. 2).

## Results and discussion

To demonstrate the utility of the transXpress pipeline, we processed RNA-seq reads from long pepper (*Piper longum*), also known as pippali, a non-model plant used in Indian Ayurvedic medicine [52]. *P.* *longum* plants have been used in traditional medicine from ancient times and are known to produce biochemically interesting alkaloids with anticancer and nootropic effects in humans [53, 54]. The RNA-seq data were downloaded from NCBI Sequence Read Archive (SRA) and contained Illumina stranded, paired-end 2 × 150 bp reads from *Piper longum* leaf, spike and root samples. The *transXpress* pipeline was run on a computational cluster with either Trinity or rnaSPADES as the assembler of choice. Notably, both de novo assemblers generated over 200 thousand unique transcripts with an average predicted ORF length of 282 and 255 amino acids, respectively (Table 2). In comparison, a recent genome assembly of the closely related black pepper (*Piper nigrum*) [55] contains 63,466 genes with the average protein coding sequence length 1347 nt (449 amino acids). This difference is likely related to the large proportion (22%) of 5′-partial transcripts, possibly caused by incomplete PCR amplification using oligo (dT) primers, as commonly performed in RNA-seq protocols. It is worth noting that for such 5′-partial protein sequences, targeting peptide prediction is not possible.

**Table 2:** Descriptive statistics of the *P. longum* transcriptomes assembled with transXpress using the Trinity and rnaSPADES assemblers

	Trinity (v2.13.2)	rnaSPADES (v3.13.0)
Number of raw sequencing reads (input data)	16,901,456 (leaf) + 22,900,035 (spike) + 27,496,748 (root) = 67,298,239 total reads	
Number of assembled transcripts (isoforms)	268,313	296,600
Number of reconstructed genes (Trinity estimate)	132,944	–
Min / median / mean / max transcript lengths	185/577/914/15,159	112/363/832/15,665
Number of predicted protein ORFs (TransDecoder)	131,098	118,984
% of full-length ORFs (TransDecoder estimate)	54.7	60.4
Min / median / mean / max ORF lengths	85/200/282/4982	85 / 191 / 255 / 5091
Transcriptome completeness (BUSCO, embryophyta_odb10 lineage)	C: 95.2% [S: 10.5%, D: 84.7%], F: 2.7%, M: 2.1%	C: 84.1% [S: 18.6%, D: 65.5%], F: 11.1%, M: 4.8%
% of reads aligned to the transcriptome (Bowtie2)	87.5%	83.3%

The estimate of the number of reconstructed genes is only generated by Trinity, by grouping the transcript isoforms that likely originated from the same gene

**Fig. 3:**
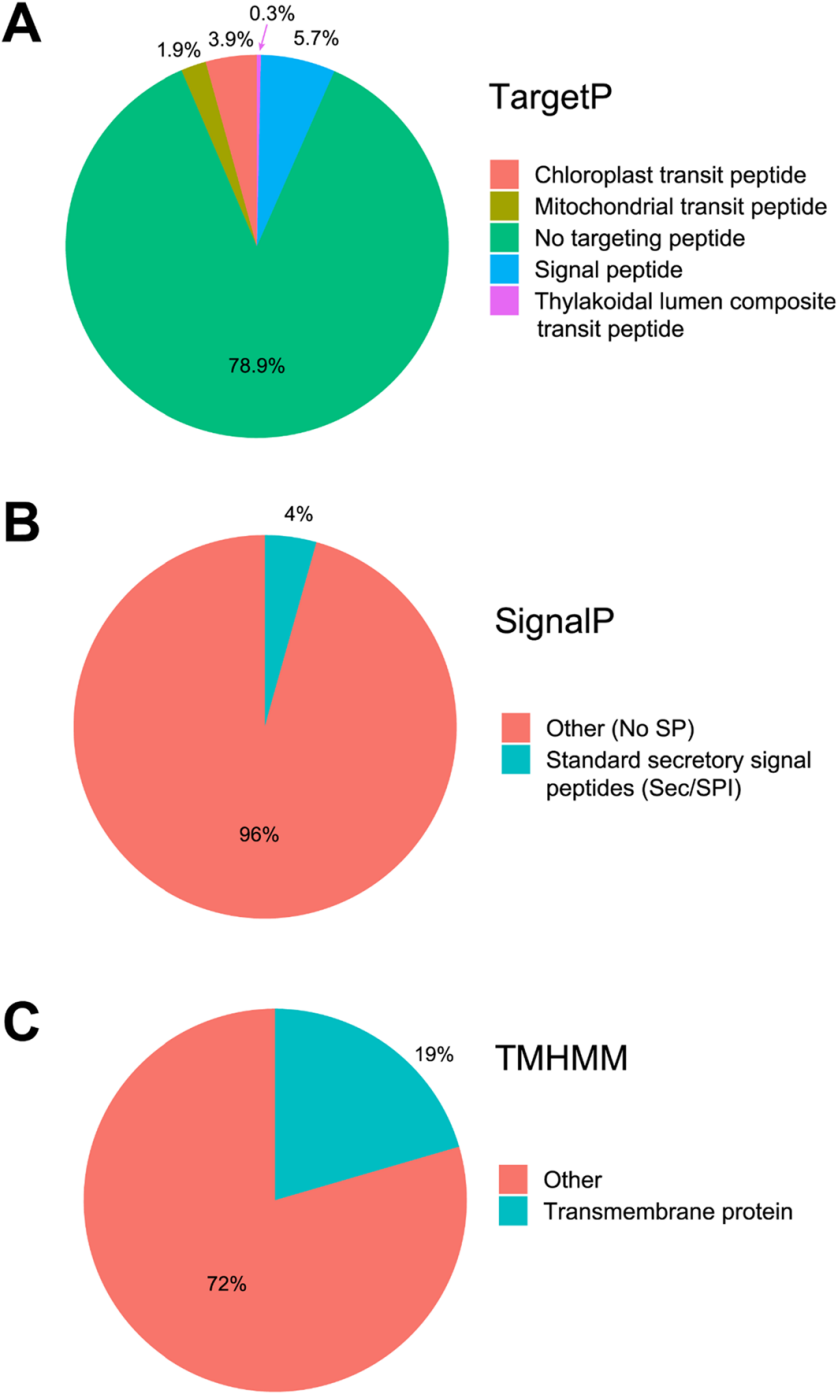
Statistics of the predicted *P.* *longum* protein sequences (n = 131,098) generated automatically using the tools included in transXpress. Data from the Trinity assembly is shown, as the results for the rnaSPADES assembly were very similar. **A** N-terminal targeting peptides predicted by TargetP. **B** N-terminal signaling peptides predicted by SignalP. **C** Transmembrane proteins predicted by TMHMM

**Fig. 4:**
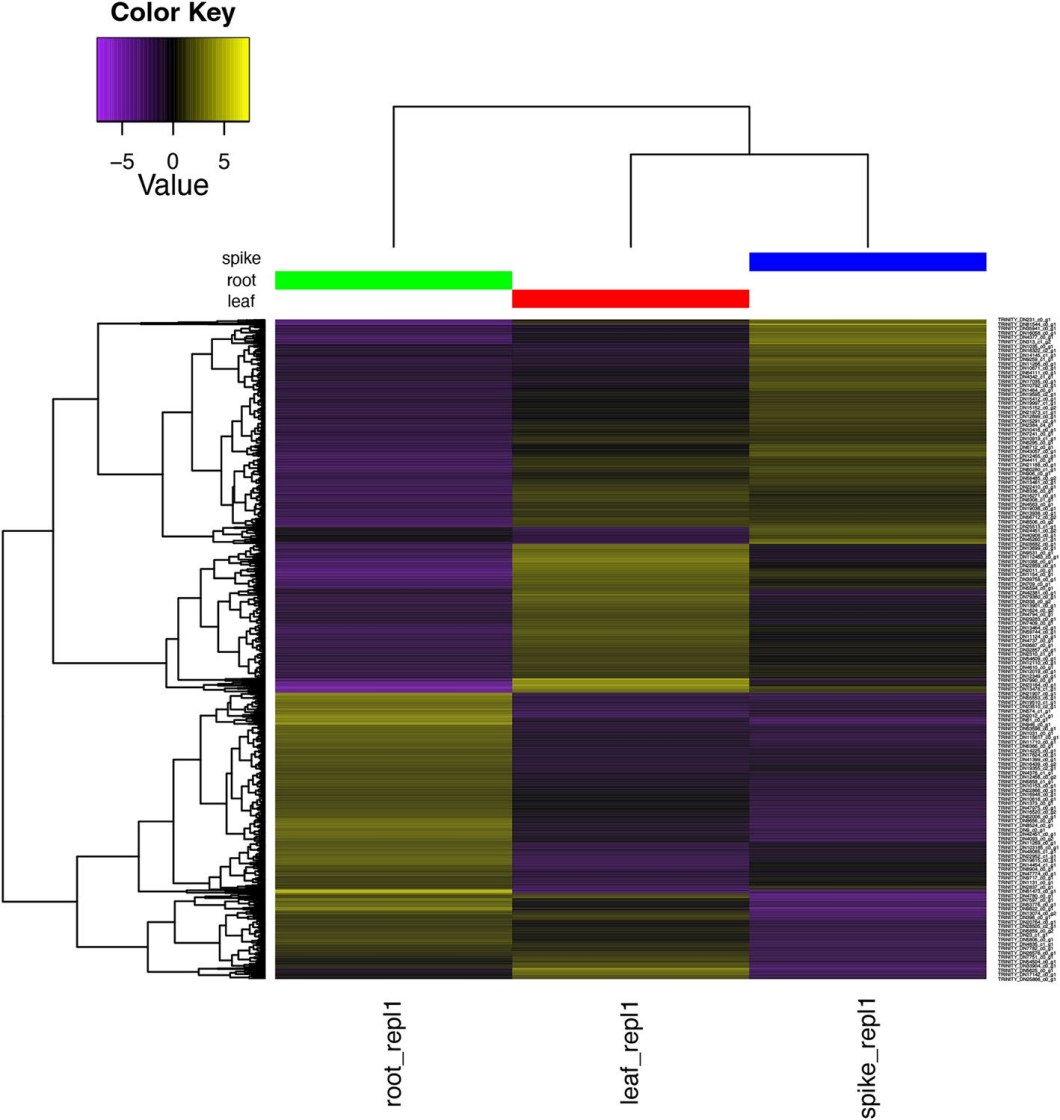
A hierarchically-clustered heatmap showing differential expression between root, leaf and tissue RNA-Seq samples from *Piper longum* [52]. This figure was automatically generated by the differential expression analysis step of transXpress from the transcriptome assembled with Trinity

Targeting peptides were found in 11.8% of the protein sequences using TargetP. The most common targeting sequence was a signal peptide for endoplasmic reticulum, followed by a chloroplast transit peptide (Fig. 3A, B). About 19% of all protein sequences were predicted to contain transmembrane domains (Fig. 3C). Differential expression analysis of the three tissue samples was performed using edgeR [40] (Fig. 4).

## Conclusions

The transXpress pipeline is an easy-to-install, integrated tool that generates reproducible, annotated FASTA files ready for downstream mining. With this, transXpress facilitates rapid discovery of new genes and proteins in non-model organisms. The pipeline is actively maintained and is already used by many labs. For experienced users, transXpress can provide a good starting point to develop customized workflows.

## Availability and requirements

Project name: transXpress.

Project home page: https://github.com/transXpress/transXpress

Operating system(s): Linux.

Programming language: Snakemake (Python), bash.

Other requirements: Dependencies installed via Conda or pip.

License: GNU GPLv3.

Any restrictions to use by non-academics: none.

## Data Availability

The datasets analyzed during the current study are available in the NCBI SRA repository, containing *Piper longum* leaf (SRR10362954), spike (SRR10362953) and root (SRR10583928) RNA-seq datasets [52]. Two archives with the output files produced by the transXpress runs using Trinity and rnaSPADES on the *Piper longum* sequencing datasets were deposited into Zenodo under https://doi.org/10.5281/zenodo.7380017 [56].
